# 4-(Methyl­sulfon­yl)benzaldehyde

**DOI:** 10.1107/S1600536809046406

**Published:** 2009-11-07

**Authors:** Shao-Song Qian, Hong-You Cui

**Affiliations:** a School of Life Sciences, ShanDong University of Technology, ZiBo 255049, People’s Republic of China; b School of Chemical Engineering, ShanDong University of Technology, ZiBo 255049, People’s Republic of China.

## Abstract

In the crystal of the title compound, C_8_H_8_O_3_S, the mol­ecules are linked into a three-dimensional array by inter­molecular C—H⋯O hydrogen bonds.

## Related literature

For reference bond-length data, see: Allen *et al.* (1987 ▶). For a related structure, see: Ma (2008 ▶). For synthetic details, see: Rivett *et al.* (1979 ▶).
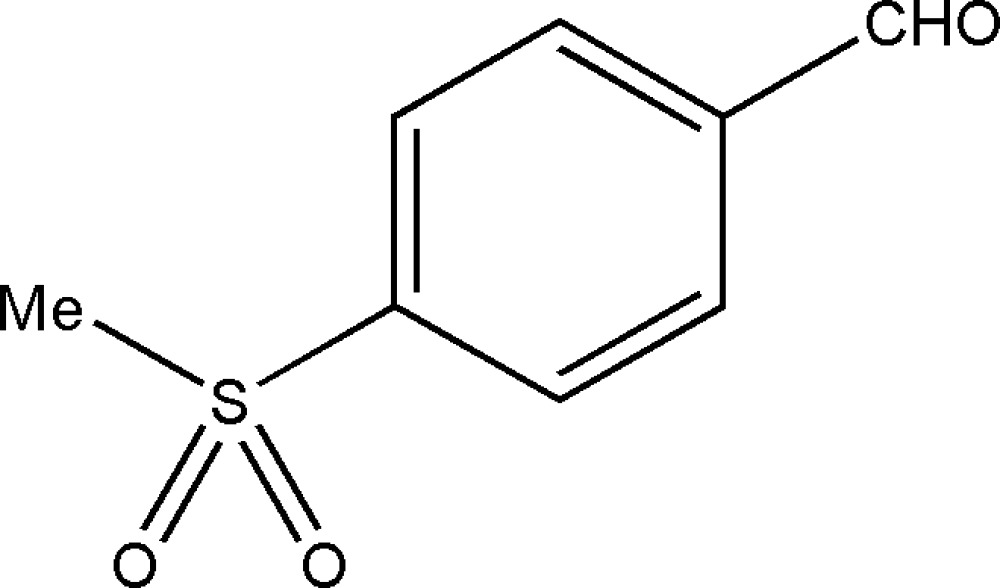


## Experimental

### 

#### Crystal data

C_8_H_8_O_3_S
*M* *_r_* = 184.20
Monoclinic, 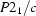

*a* = 6.1280 (12) Å
*b* = 8.0400 (16) Å
*c* = 16.734 (3) Å
β = 90.07 (3)°
*V* = 824.5 (3) Å^3^
*Z* = 4
Mo *K*α radiation
μ = 0.35 mm^−1^
*T* = 293 K
0.30 × 0.20 × 0.20 mm

#### Data collection

Enraf–Nonius CAD-4 diffractometer
Absorption correction: multi-scan (*SADABS*; Sheldrick, 1996 ▶) *T*_min_ = 0.902, *T*_max_ = 0.933
1643 measured reflections
1495 independent reflections
1310 reflections with *I* > 2σ(*I*)
*R*_int_ = 0.013
3 standard reflections every 200 reflections intensity decay: 1%

#### Refinement

*R*[*F* ^2^ > 2σ(*F* ^2^)] = 0.034
*wR*(*F* ^2^) = 0.126
*S* = 1.00
1495 reflections
110 parameters
H-atom parameters constrained
Δρ_max_ = 0.22 e Å^−3^
Δρ_min_ = −0.26 e Å^−3^

### 

Data collection: *CAD-4 Software* (Enraf–Nonius 1989 ▶); cell refinement: *CAD-4 Software*; data reduction: *XCAD4* (Harms & Wocadlo,1995 ▶); program(s) used to solve structure: *SHELXS97* (Sheldrick, 2008 ▶); program(s) used to refine structure: *SHELXL97* (Sheldrick, 2008 ▶); molecular graphics: *SHELXTL* (Sheldrick, 2008 ▶); software used to prepare material for publication: *SHELXTL*.

## Supplementary Material

Crystal structure: contains datablocks global, I. DOI: 10.1107/S1600536809046406/wn2364sup1.cif

Structure factors: contains datablocks I. DOI: 10.1107/S1600536809046406/wn2364Isup2.hkl

Additional supplementary materials:  crystallographic information; 3D view; checkCIF report

## Figures and Tables

**Table 1:** Hydrogen-bond geometry (Å, °)

*D*—H⋯*A*	*D*—H	H⋯*A*	*D*⋯*A*	*D*—H⋯*A*
C4—H4*A*⋯O3^i^	0.93	2.57	3.457 (3)	159
C1—H1*D*⋯O1^ii^	0.96	2.56	3.518 (3)	176

Symmetry codes: (i) 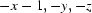; (ii) 
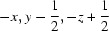
.

**Fig. 1.**
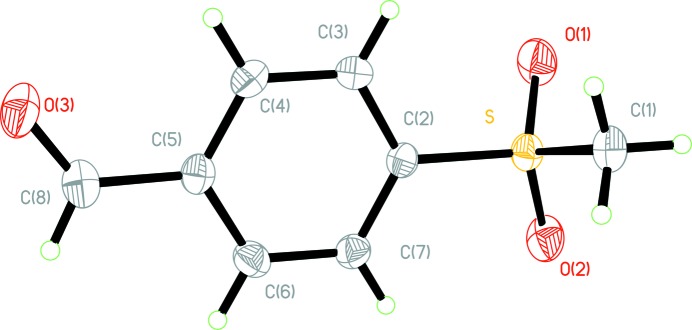
The molecular structure of the title compound, showing the atom-labelling scheme. Displacement ellipsoids are drawn at the 35% probability level. Hydrogen atoms are shown as small spheres of arbitrary radius.

**Fig. 2.**
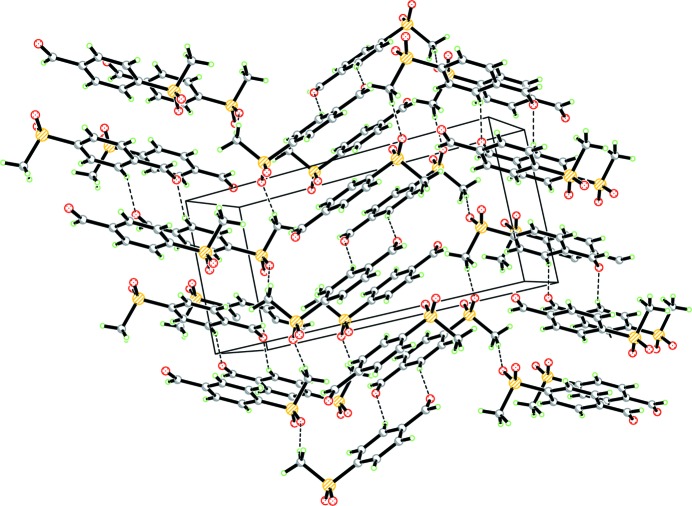
The crystal packing of the title compound. Dashed lines indicate hydrogen bonds.

## supplementary crystallographic information

### Comment

The title compound has been of great of interest for many years. It acts as an important precursor for the synthesis of amino alcohols with applications to the synthesis of the antibiotics chloramphenicol, fluoramphenicol and thiamphenicol. Here we report its crystal structure.

In the title compound (Fig. 1), all bond lengths are within normal ranges (Allen *et al.*, 1987) and comparable to the values observed in a closely related compound (Ma, 2008). The C1—S—C2—C3 torsion angle is 75.07 (17)°.

In the crystal structure, molecules are linked through intermolecular C—H···O hydrogen bonds (Table 1; Fig. 2).

### Experimental

The title compound was synthesized according to a literature method (Rivett *et al.*, 1979). 0.1 g of the title compound was dissolved in acetonitrile (20 ml). Single crystals suitable for X-ray diffraction were obtained by spontaneous evaporation of the solvent.

### Refinement

All H atoms were placed in geometrical positions and constrained to ride on their parent atoms with C—H distances in the range 0.93–0.96 Å, They were treated as riding atoms, with *U*_iso_(H) = k*U*_eq_(C), where k = 1.5 for methyl and 1.2 for all other H atoms.

### Crystal data

**Table:**

C_8_H_8_O_3_S	*F*(000) = 384
*M**_r_* = 184.20	*D*_x_ = 1.484 Mg m^−^^3^
Monoclinic, *P*2_1_/*c*	Mo *K*α radiation, λ = 0.71073 Å
*a* = 6.1280 (12) Å	Cell parameters from 25 reflections
*b* = 8.0400 (16) Å	θ = 9–13°
*c* = 16.734 (3) Å	µ = 0.35 mm^−^^1^
β = 90.07 (3)°	*T* = 293 K
*V* = 824.5 (3) Å^3^	Block, yellow
*Z* = 4	0.30 × 0.20 × 0.20 mm

### Data collection

**Table:**

Enraf–Nonius CAD-4 diffractometer	1310 reflections with *I* > 2σ(*I*)
Radiation source: fine-focus sealed tube	*R*_int_ = 0.013
graphite	θ_max_ = 25.2°, θ_min_ = 2.4°
ω/2θ scans	*h* = −7→0
Absorption correction: multi-scan (*SADABS*; Sheldrick, 1996)	*k* = −9→0
*T*_min_ = 0.902, *T*_max_ = 0.933	*l* = −20→20
1643 measured reflections	3 standard reflections every 200 reflections
1495 independent reflections	intensity decay: 1%

### Refinement

**Table:**

Refinement on *F*^2^	Secondary atom site location: difference Fourier map
Least-squares matrix: full	Hydrogen site location: inferred from neighbouring sites
*R*[*F*^2^ > 2σ(*F*^2^)] = 0.034	H-atom parameters constrained
*wR*(*F*^2^) = 0.126	*w* = 1/[σ^2^(*F*_o_^2^) + (0.1*P*)^2^ + 0.095*P*] where *P* = (*F*_o_^2^ + 2*F*_c_^2^)/3
*S* = 1.00	(Δ/σ)_max_ < 0.001
1495 reflections	Δρ_max_ = 0.22 e Å^−^^3^
110 parameters	Δρ_min_ = −0.26 e Å^−^^3^
0 restraints	Extinction correction: *SHELXL97* (Sheldrick, 2008), Fc^*^=kFc[1+0.001xFc^2^λ^3^/sin(2θ)]^-1/4^
Primary atom site location: structure-invariant direct methods	Extinction coefficient: 0.028 (6)

### Special details

**Table:**

Geometry. All esds (except the esd in the dihedral angle between two l.s. planes) are estimated using the full covariance matrix. The cell esds are taken into account individually in the estimation of esds in distances, angles and torsion angles; correlations between esds in cell parameters are only used when they are defined by crystal symmetry. An approximate (isotropic) treatment of cell esds is used for estimating esds involving l.s. planes.
Refinement. Refinement of F^2^ against ALL reflections. The weighted R-factor wR and goodness of fit S are based on F^2^, conventional R-factors R are based on F, with F set to zero for negative F^2^. The threshold expression of F^2^ > 2sigma(F^2^) is used only for calculating R-factors(gt) etc. and is not relevant to the choice of reflections for refinement. R-factors based on F^2^ are statistically about twice as large as those based on F, and R- factors based on ALL data will be even larger.

### Fractional atomic coordinates and isotropic or equivalent isotropic displacement parameters (Å^2^)

**Table:**

	*x*	*y*	*z*	*U*_iso_*/*U*_eq_	
S	0.25890 (8)	0.43803 (6)	0.17148 (3)	0.0397 (3)	
O1	0.1031 (3)	0.5283 (2)	0.21856 (10)	0.0647 (5)	
O2	0.4429 (3)	0.5269 (2)	0.13956 (10)	0.0590 (5)	
C1	0.3533 (4)	0.2698 (3)	0.22913 (12)	0.0479 (5)	
H1B	0.4308	0.3111	0.2749	0.072*	
H1C	0.4492	0.2022	0.1975	0.072*	
H1D	0.2314	0.2041	0.2465	0.072*	
C2	0.1206 (3)	0.3429 (2)	0.09056 (10)	0.0338 (4)	
C3	−0.0839 (3)	0.2728 (3)	0.10317 (12)	0.0436 (5)	
H3A	−0.1484	0.2768	0.1534	0.052*	
O3	−0.3837 (3)	0.0537 (2)	−0.10080 (11)	0.0631 (5)	
C4	−0.1897 (3)	0.1971 (3)	0.04005 (12)	0.0427 (5)	
H4A	−0.3256	0.1482	0.0477	0.051*	
C5	−0.0929 (3)	0.1941 (2)	−0.03484 (11)	0.0373 (5)	
C6	0.1115 (4)	0.2644 (3)	−0.04627 (12)	0.0432 (5)	
H6A	0.1757	0.2613	−0.0966	0.052*	
C7	0.2200 (3)	0.3388 (2)	0.01639 (11)	0.0392 (5)	
H7A	0.3575	0.3854	0.0090	0.047*	
C8	−0.2049 (4)	0.1147 (3)	−0.10336 (13)	0.0504 (6)	
H8A	−0.1315	0.1119	−0.1520	0.061*	

### Atomic displacement parameters (Å^2^)

**Table:**

	*U*^11^	*U*^22^	*U*^33^	*U*^12^	*U*^13^	*U*^23^
S	0.0415 (4)	0.0387 (4)	0.0387 (4)	−0.00006 (19)	−0.0065 (2)	−0.00429 (18)
O1	0.0675 (11)	0.0680 (11)	0.0586 (10)	0.0206 (9)	−0.0087 (9)	−0.0250 (8)
O2	0.0628 (10)	0.0582 (9)	0.0560 (9)	−0.0261 (8)	−0.0096 (8)	0.0025 (8)
C1	0.0512 (12)	0.0505 (12)	0.0421 (11)	0.0021 (10)	−0.0120 (9)	0.0027 (9)
C2	0.0330 (9)	0.0330 (9)	0.0352 (9)	0.0010 (7)	−0.0039 (7)	0.0013 (7)
C3	0.0365 (10)	0.0567 (12)	0.0374 (10)	−0.0024 (9)	0.0051 (8)	−0.0022 (9)
O3	0.0617 (11)	0.0626 (10)	0.0649 (11)	−0.0178 (8)	−0.0167 (8)	−0.0050 (8)
C4	0.0331 (10)	0.0461 (11)	0.0488 (11)	−0.0050 (8)	−0.0015 (8)	0.0001 (8)
C5	0.0398 (10)	0.0323 (9)	0.0396 (10)	0.0018 (8)	−0.0072 (8)	0.0008 (7)
C6	0.0472 (11)	0.0486 (11)	0.0338 (10)	−0.0028 (9)	0.0041 (8)	0.0009 (8)
C7	0.0352 (10)	0.0432 (10)	0.0391 (10)	−0.0045 (8)	0.0005 (8)	0.0030 (8)
C8	0.0595 (14)	0.0469 (12)	0.0448 (11)	−0.0042 (11)	−0.0090 (10)	−0.0014 (9)

### Geometric parameters (Å, °)

**Table:**

S—O1	1.4355 (17)	C3—H3A	0.9300
S—O2	1.4385 (17)	O3—C8	1.201 (3)
S—C1	1.759 (2)	C4—C5	1.387 (3)
S—C2	1.7703 (18)	C4—H4A	0.9300
C1—H1B	0.9600	C5—C6	1.388 (3)
C1—H1C	0.9600	C5—C8	1.480 (3)
C1—H1D	0.9600	C6—C7	1.377 (3)
C2—C7	1.384 (3)	C6—H6A	0.9300
C2—C3	1.390 (3)	C7—H7A	0.9300
C3—C4	1.380 (3)	C8—H8A	0.9300
			
O1—S—O2	118.34 (12)	C2—C3—H3A	120.5
O1—S—C1	107.85 (11)	C3—C4—C5	119.86 (18)
O2—S—C1	109.17 (11)	C3—C4—H4A	120.1
O1—S—C2	108.66 (10)	C5—C4—H4A	120.1
O2—S—C2	107.77 (9)	C4—C5—C6	120.28 (18)
C1—S—C2	104.13 (9)	C4—C5—C8	120.61 (18)
S—C1—H1B	109.5	C6—C5—C8	119.11 (19)
S—C1—H1C	109.5	C7—C6—C5	120.46 (18)
H1B—C1—H1C	109.5	C7—C6—H6A	119.8
S—C1—H1D	109.5	C5—C6—H6A	119.8
H1B—C1—H1D	109.5	C6—C7—C2	118.71 (17)
H1C—C1—H1D	109.5	C6—C7—H7A	120.6
C7—C2—C3	121.62 (17)	C2—C7—H7A	120.6
C7—C2—S	119.04 (14)	O3—C8—C5	124.8 (2)
C3—C2—S	119.34 (14)	O3—C8—H8A	117.6
C4—C3—C2	119.05 (18)	C5—C8—H8A	117.6
C4—C3—H3A	120.5		
			
O1—S—C2—C7	140.91 (17)	C3—C4—C5—C6	1.1 (3)
O2—S—C2—C7	11.52 (19)	C3—C4—C5—C8	−179.47 (18)
C1—S—C2—C7	−104.34 (17)	C4—C5—C6—C7	−0.4 (3)
O1—S—C2—C3	−39.78 (18)	C8—C5—C6—C7	−179.85 (18)
O2—S—C2—C3	−169.16 (16)	C5—C6—C7—C2	−0.4 (3)
C1—S—C2—C3	74.97 (17)	C3—C2—C7—C6	0.6 (3)
C7—C2—C3—C4	0.1 (3)	S—C2—C7—C6	179.91 (14)
S—C2—C3—C4	−179.23 (15)	C4—C5—C8—O3	2.4 (3)
C2—C3—C4—C5	−0.9 (3)	C6—C5—C8—O3	−178.2 (2)

### Hydrogen-bond geometry (Å, °)

**Table:**

*D*—H···*A*	*D*—H	H···*A*	*D*···*A*	*D*—H···*A*
C4—H4A···O3^i^	0.93	2.57	3.457 (3)	159
C1—H1D···O1^ii^	0.96	2.56	3.518 (3)	176

Symmetry codes: (i) −*x*−1, −*y*, −*z*; (ii) −*x*, *y*−1/2, −*z*+1/2.
